# RTK_RAG: Leveraging Retrieval Augmented Generation with Multi-Window Convolutional Neural Networks for Superior ATP Binding Site Prediction in Receptor Tyrosine Kinases

**DOI:** 10.1021/acs.jcim.5c00766

**Published:** 2025-06-18

**Authors:** Sin-Siang Wei, Wei-En Jhang, Yu-Chen Liu, Cheng-Che Chuang, Yu-Yen Ou

**Affiliations:** † Department of Computer Science and Engineering, 34895 Yuan Ze University, Chung-Li 32003, Taiwan; ‡ Graduate Program in Biomedical Informatics, Yuan Ze University, Chung-Li 32003, Taiwan

## Abstract

Receptor tyrosine kinases (RTKs) are key regulators of cellular signaling and are frequently involved in cancer development. As their activation depends on ATP binding to the kinase domain, precisely identifying ATP binding sites is critical for mechanistic studies and targeted therapy development. However, general ATP binding site prediction methods often fall short for RTKs due to their diverse structural features across different protein families. To address this challenge, we introduce RTK_RAG, a framework that integrates retrieval-augmented generation (RAG) and utilizes protein language models (PLMs) with a multiwindow convolutional neural network (MCNN) architecture to improve ATP binding site prediction for RTKs. When tested on an independent RTK data set, RTK_RAG outperforms general ATP binding site predictors on multiple evaluation metrics. By accounting for RTK-specific structural differences, our study provides a reliable tool for researching RTK function and facilitating the development of novel kinase inhibitors. Moreover, this approach demonstrates the potential of RAG-based frameworks for enhancing functional predictions in specialized protein families, offering a generalizable strategy for improving binding site identification in specific protein families.

## Graphical abstract

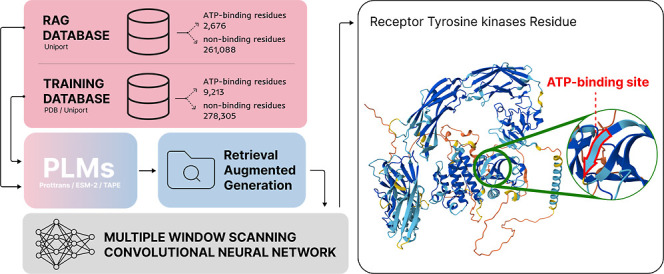

## Introduction

Receptor tyrosine kinases (RTKs) constitute a family of transmembrane proteins that pivotally regulate essential cellular processes, including growth, differentiation, and metabolism.^1^ Their aberrant activity is a well-established driver of cancer initiation and progression, positioning RTKs as key targets for anticancer drug development.^2^ Furthermore, the pursuit of effective kinase-inhibitor scaffolds remains a central theme in medicinal chemistry. Among these scaffolds, pyrimidine-based compounds^3^ have emerged as particularly promising candidates for tyrosine kinase inhibition, thereby expanding the therapeutic arsenal against cancer.

As illustrated in Figure 1, RTK activation is intrinsically dependent on the binding of adenosine triphosphate (ATP) to their intracellular kinase domain. The ATP binding facilitates subsequent autophosphorylation of tyrosine residues, initiating downstream signaling cascades that, when dysregulated, can promote cancer progression.^4^ Consequently, the precise identification and characterization of these ATP binding sites are indispensable for both fundamental mechanistic studies and the rational design of targeted therapies, including potent and selective receptor tyrosine kinase inhibitors (RTKIs). Insights from advances in inhibitors targeting other tyrosine kinases, such as Bruton’s tyrosine kinase inhibitors (BTKIs), also underscore the importance of understanding the molecular and structural basis of ATP-binding site recognition across the broader kinase family.^5^ These findings and the critical role of RTK both emphasize the necessity of high-quality molecular mapping of ATP-binding pockets for inhibitor design and optimization.

**Figure 1:**
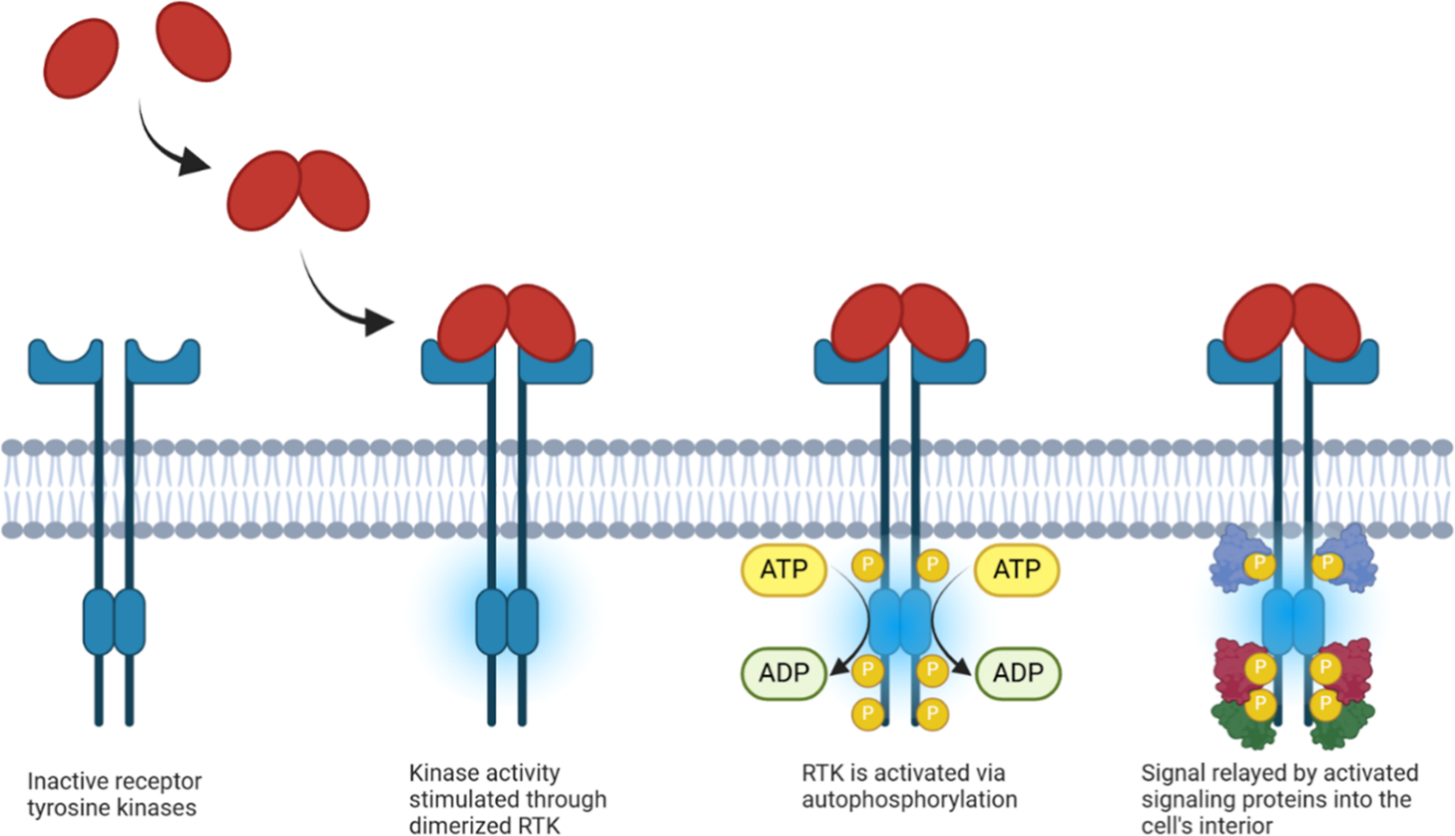
Activation of receptor tyrosine kinases.

Precise modeling and prediction of ATP-binding site structure enable researchers to design inhibitors that can selectively block RTK activity and halt disease progression.^6^ Figure 2 illustrates Erlotinib, a clinically approved EGFR inhibitor, functions competitively by binding to the ATP-binding pocket of the kinase in its active conformation.^7,8^ The prediction of ATP-binding sites is also fundamental to advancing targeted therapies^9,10^ for addressing drug resistance associated with cancer and other RTK-driven diseases. Notably, the ongoing development of kinase inhibitors with potentially superior potency to established agents like Erlotinib highlights the critical role of accurate ATP-binding site prediction in uncovering novel therapeutic avenues and optimizing drug–target interactions.^3^ This targeted strategy not only increases the potential to reduce off-target effects and improve overall drug efficacy but also contributes to the development of next-generation inhibitors that are capable of overcoming resistance arising from mutations or altered binding site conformations.^11,12^

**Figure 2:**
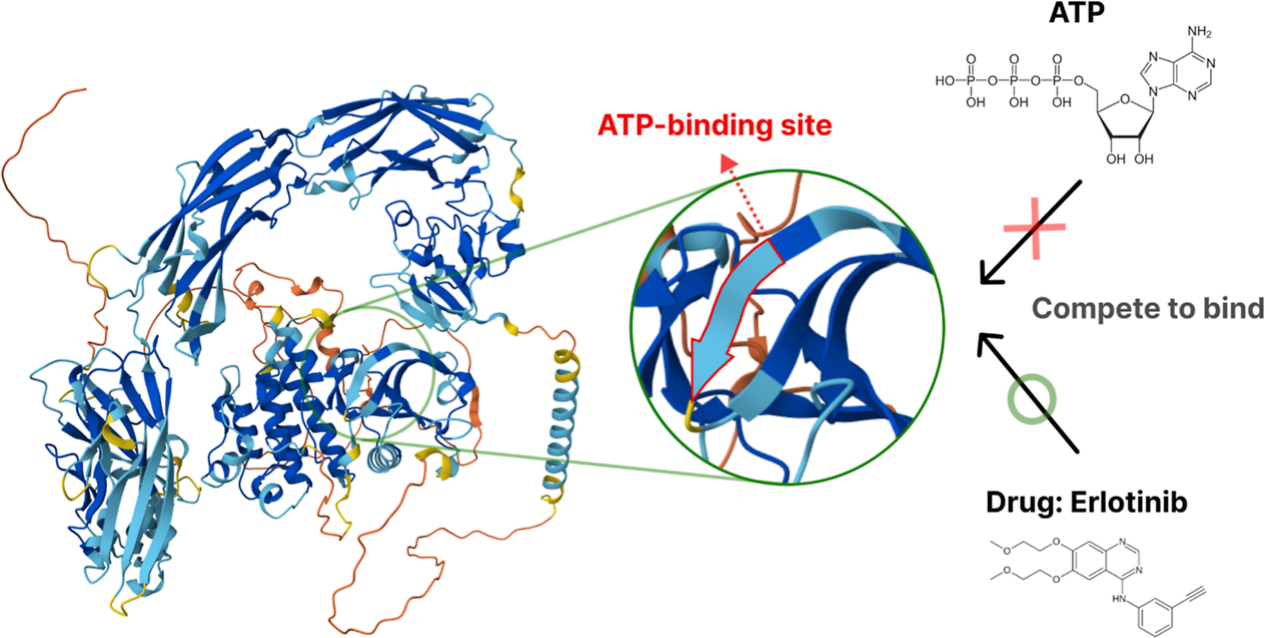
Competitive binding mechanism of Erlotinib (Type I RTKI) to the ATP-binding site of RTKs.

Despite this critical need, a significant challenge persists: while existing general ATP binding site prediction methods may be useful for many protein families, they often struggle to achieve high accuracy when applied specifically to RTKs. This limitation primarily stems from the inherent structural and sequence diversity across RTK families, whose unique features distinguish members from one another. These key features are frequently overlooked by generic prediction tools, leading to inaccuracies that can misdirect drug discovery efforts. This deficiency directly impedes the efficient discovery and development of novel RTKIs.

To address this gap and enhance the accuracy of ATP binding site prediction for the diverse and therapeutically important RTK superfamily, we introduce RTK_RAG. This novel computational framework uniquely integrates the strengths of retrieval-augmented generation (RAG) with advanced protein language models (PLMs) and a multiwindow convolutional neural network (MCNN) architecture. Protein language models, such as ProtTrans^13^ and ESM-2,^14^ have marked a breakthrough in protein sequence analysis by learning rich representations from vast amounts of protein data, thereby substantially improving binding site prediction capabilities by effectively capturing complex biological signals within amino acid sequences.^15,16^ While PLMs excel at capturing global sequence patterns, their analyses may overlook local spatial features critical for precise binding site recognition. To complement this, MCNNs are employed to extract such multiscale spatial information^17^ from protein sequence embeddings by analyzing them through varying window sizes, thus capturing both local motifs and broader contextual features relevant for identifying functional sites like ATP-binding pockets.^18^

Recognizing that relying solely on sequence information from a specific protein family may not sufficiently capture all its defining characteristics,^13^ RTK_RAG incorporates a RAG strategy.^19^ This approach leverages an external knowledge base, constructed from multiple protein families known to bind ATP, to enrich the model’s understanding of RTK structure and function. By retrieving and integrating relevant information from this curated database, RTK_RAG gains the ability to predict ATP-binding sites with greater precision, particularly for subtle binding site recognition within RTKs.

By synergizing the global understanding of PLMs, the local feature extraction of MCNNs, and the context-enriching power of RAG, RTK_RAG offers a specialized and robust tool. Our study demonstrates that this tailored approach significantly outperforms general ATP binding site predictors for RTKs, providing a reliable method for researching RTK function and facilitating the development of novel kinase inhibitors. This work not only provides a valuable tool for the RTK field but also illustrates the potential of RAG-based frameworks for enhancing functional predictions in other specialized protein families.

## Materials and Methods

The overall workflow for our study is illustrated in Figure 3. Initially, we collected protein sequences from the UniProt and recently constructed data sets to ensure diverse data for training and validation. Subsequently, protein sequences are transformed into high-dimensional embeddings using a pretrained protein language model, such as ProtTrans, to capture essential biological signals. These embeddings are then analyzed by a multiwindow scanning convolutional neural network (MCNN) combined with RAG that integrates key external biological information. Residues within proteins were classified into ATP-binding or nonbinding categories, and the predictive performance of the model was rigorously validated using an independent RTK data set. This workflow underscores how effectively combining protein language models, deep learning techniques, and RAG can advance protein function prediction, particularly in identifying ATP-binding residues.

**Figure 3:**
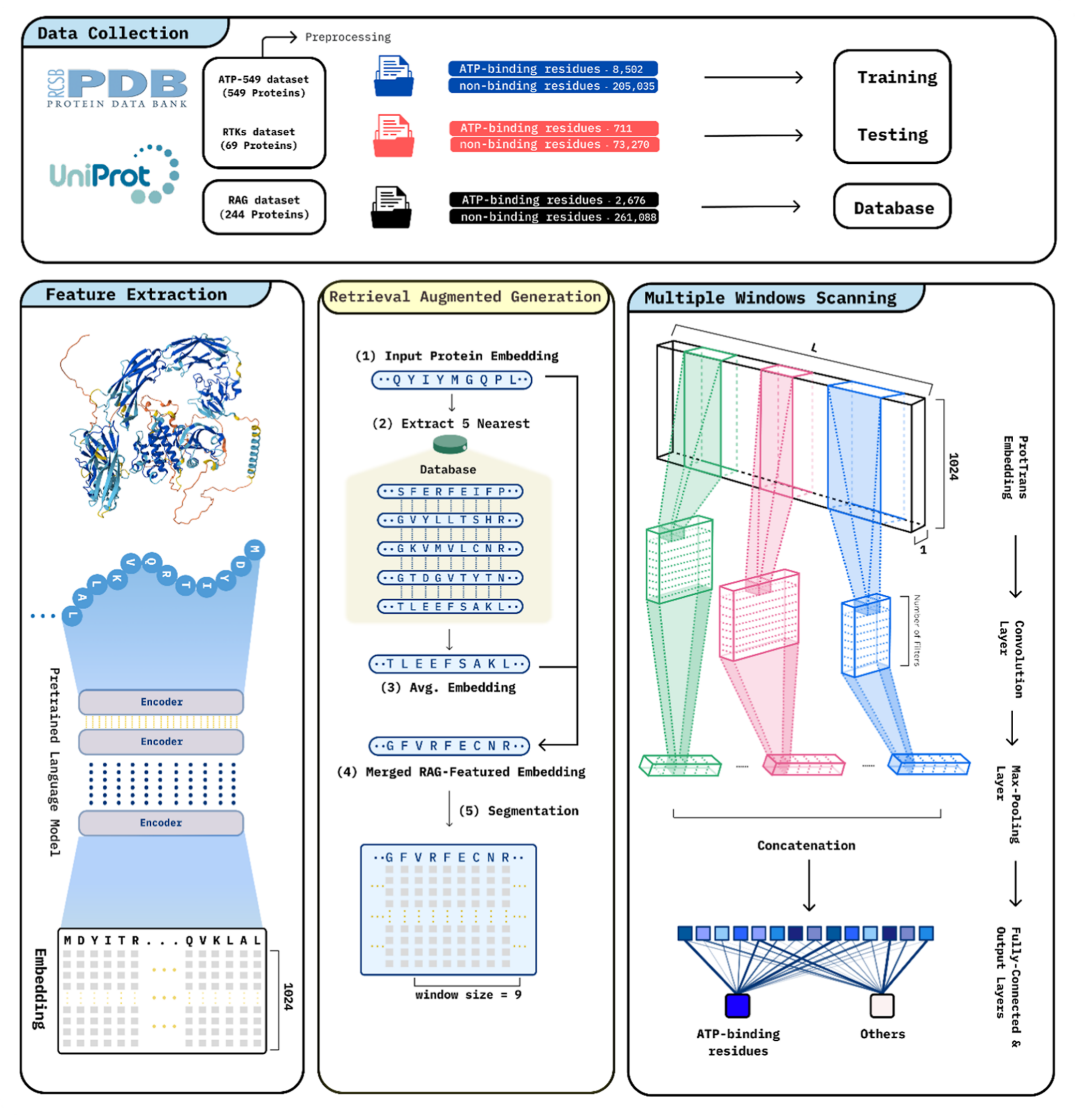
Overall workflow of RTK_RAG for predicting ATP-binding sites in RTKs.

### Data Collection

The final composition of our resources is presented in Table 1. Due to the limited availability of RTK-specific protein sequences for model training, we selected the ATP-549 data set as our primary training resource. ATP-549, a newly constructed data set from the ATP-Deep predictor,^20^ was developed to address limitations associated with small and low-diversity protein data sets. It contains 549 protein sequences, comprising 8502 ATP-binding residues and 205,035 nonbinding residues. By utilizing this complete biological information provided by ATP-549, we aim to overcome the scarcity of RTK-specific sequences and effectively train our prediction model.

**Table 1:** Statistics of the Dataset

data set	protein sequences	similarity <40%
ATP-549	549	549
RTKs	313	69

data set	protein sequences	ATP binding residues	non-binding residues
ATP-549 (train)	549	8502	205,035
RTKs (independent test)	69	711	73,270
RAG database	244	2676	261,088

Concurrently, we collected additional 313 RTK sequences from UniProt to build the foundation of our external retrieval-augmented generation (RAG) database. To prevent excessive similarity between ATP-549 and the newly gathered RTK(313) set, we performed a single run of CD-HIT^21^ at a 40% sequence identity threshold on both data sets. This step reduced redundancy and yielded 69 RTK proteins, which were designated for validation, while the remaining 244 sequences formed the final RAG database that comprising 2676 ATP-binding residues and 261,088 nonbinding residues.

### Embedding Generation with Pre-trained Protein Language Models

Protein language models (PLMs) generate high-dimensional embeddings that capture contextual dependencies and subtle sequence variations, making them highly suited for complex biological prediction tasks.^22^ In this study, we employed ProtTrans,^13^ an advanced PLM built on the Transformer architecture to generate embeddings for extracting key features in the amino acid sequences. ProtTrans is pretrained on the large-scale UniRef50 data set, allowing it to capture conserved features across diverse protein families. The embeddings produced by ProtTrans (up to 1024 dimensions) provide a rich representation of both sequence-level information and higher-order structural and functional features. By capitalizing on the contextual information encoded in these embeddings, ProtTrans enables precise identification of functional and interaction sites. This makes it particularly effective for predicting complex biological functions compared to traditional alignment-based approaches.

### Retrieval-Augmented Generation (RAG) Framework for Protein Sequence Representation

Large language models (LLMs) have achieved significant breakthroughs in natural language processing (NLP) tasks in recent years, mainly due to their ability to derive deep contextual understanding from extensive data sets. A key advancement enabling this progress is the retrieval-augmented generation (RAG) framework, which enhances LLMs by integrating relevant information from external databases into their outputs.^23,24^

As illustrated in Figure 4, the RAG framework begins with an input query that is transformed into an embedding. This embedding is compared against a prebuilt vector store containing relevant sequences or data. The system retrieves the most similar sequences, providing valuable contextual information. These retrieved sequences are then combined with the original query to form an enriched prompt, which is passed into an LLM. The LLM generates output based on both the original input and the retrieved contextual data. By fusing stored knowledge with retrieval, the RAG framework significantly improves the accuracy and reliability of various tasks.

**Figure 4:**
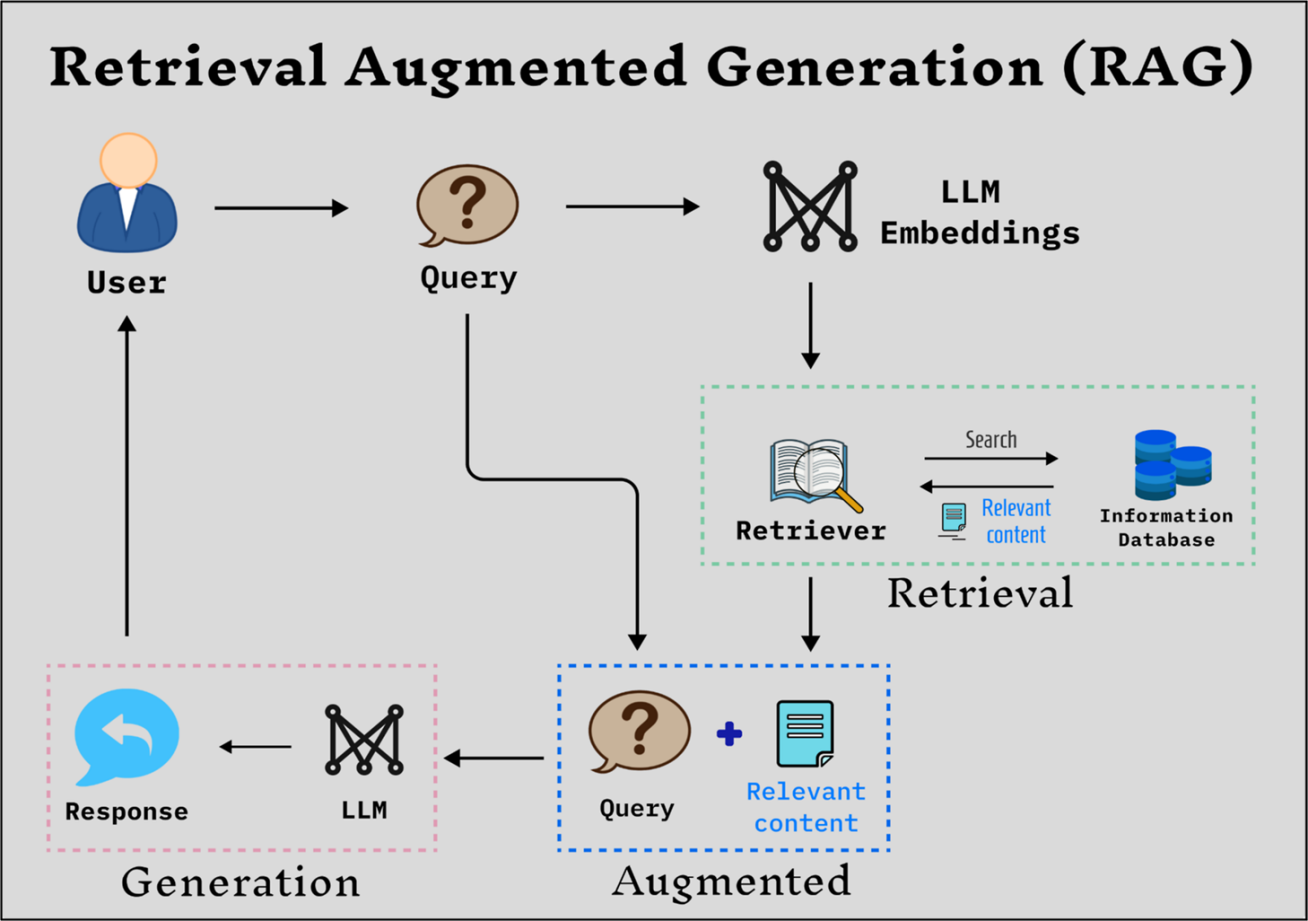
Overview of the general retrieval-augmented generation (RAG) framework.

In this work, when given a target RTK protein sequence, we first convert it into a feature vector using a protein language model (e.g., ProtTrans), which outputs an *L* × 1 × 1024 embedding, where “*L*” is the sequence length. This embedding reflects global contextual information, enabling a nuanced analysis of the protein’s biochemical features. Next, we compare this embedding against a database of precomputed embeddings using a distance metric to search for the top K similar protein sequences. To balance the unique characteristics of the input sequence with the external information, we then average the embeddings of these retrieved sequences to form a contextual embedding and combine it with the original query embedding at a 1:1 ratio. This retrieval-augmented process substantially enhances the model’s ability to detect subtle differences in protein function, thereby improving the accuracy and reliability of tasks like binding site identification. By uniting retrieval and augmenting within the embedding framework, the overall sequence representation becomes more comprehensive, capturing a broader range of information that refines predictive performance.

### Multiple Window Scanning Deep Learning Networks Architecture

To extract features at multiple scales from protein sequence embeddings, we employed a multiple window scanning approach, an advanced extension of convolutional neural networks (CNNs). This technique involves sliding windows of varying sizes across the sequence embeddings to capture local patterns and features.^25,26^ For each window size, the MCNN produces a vector representation that reflects the sequence’s structural and functional context at that scale. To ensure the retention of the most important information, max-pooling was applied to the vectors generated for each window for distilling. These pooled features from all window sizes were then concatenated to form an overall feature representation, which was passed through a fully connected layer for final predictions. Through systematic experimentation, we found out that the combination of window sizes 10, 12, and 8 yielded the highest performance of this multiscale approach for modeling the diversity of protein sequences.

## Results

### The Performance Comparison with Different Features

We first investigated how different feature embeddings influenced the performance of ATP binding site prediction. Table 2 displays the results of four embedding models: ProtTrans,^13^ ESM-2 (1280 dimension), ESM-2 (2560 dimension),^14^ and TAPE.^27^ Among these, ProtTrans delivered the highest AUC (0.9497), highlighting its strong ability to capture information within protein sequences. Although ESM-2 performed competitively, particularly at a 2560-dimensional embedding, it demanded roughly twice the computational resources and time investment compared to ProtTrans. Hence, while ESM-2 offers robust sensitivity and specificity, ProtTrans stands out as the more balanced choice to provide superior AUC performance with greater efficiency. In contrast, TAPE lagged, yielding an AUC of 0.8300 and indicating limited capacity to model the intricacies of ATP-binding contexts.

**Table 2:** Performance Comparison with Different Features without RAG

feature sets	sensitivity	specificity	accuracy	MCC	AUC
ProtTrans	0.8495	0.9443	0.9434	0.3181	0.9497
ESM-2 (1280 dimension)	0.8636	0.8907	0.8904	0.2293	0.9403
ESM-2 (2560 dimension)	0.8692	0.9693	0.9683	0.4235	0.9378
TAPE	0.8031	0.7107	0.7116	0.1100	0.8300

Building upon these findings, we chose ProtTrans as our primary protein language model (PLM) due to its favorable balance of predictive performance and computational efficiency. ProtTrans facilitates the retrieval of relevant external knowledge and coherent context by offering richer protein representations, ultimately improving binding site identification in RTKs.

### The Performance Comparison with Different Splitting Window Lengths

After evaluating the performance of different features, we evaluated the performance of different splitting window lengths to determine whether adjusting the window length would enhance the model’s overall performance. As shown in Table 3, the impact of varying window lengths on model performance revealed that a window length of 9 achieved the highest AUC value of 0.9696. Both shorter and longer windows resulted in lower AUC values. For example, a window length of 8 achieved an AUC of 0.9393, while lengths of 10 and 11 dropped further to 0.9574 and 0.9385, respectively. These results indicate that excessively short windows may miss the broader context, while longer windows might introduce redundant or irrelevant information that dilutes the predictive features. Therefore, determining the appropriate window lengths is crucial to ensure the model captures relevant patterns without being overwhelmed by unnecessary information, ultimately enhancing predictive accuracy.

**Table 3:** Performance Comparison with Different Splitting Window Length^a^

window length	sensitivity	specificity	accuracy	MCC	AUC
6	0.9072	0.9239	0.9238	0.2922	0.9668
7	0.8411	0.9226	0.9218	0.2676	0.9519
8	0.8678	0.8835	0.8833	0.2226	0.9393
**9**	**0.9269**	**0.9195**	**0.9195**	**0.2905**	**0.9696**
10	0.9030	0.8914	0.8915	0.2418	0.9574
11	0.8200	0.9203	0.9194	0.2565	0.9385
12	0.8805	0.9009	0.9007	0.2471	0.9502

a The bolded row indicates the configuration selected for the final model.

### The Performance Comparison with Different Dropout Rate

The impact of different dropout rates on the model’s performance was then evaluated. Based on the number of dropout rates adjusted between 0 and 0.9, Table 4 shows how the model’s performance changes. Increasing dropout rates generally helps reduce overfitting, resulting in a more stable model performance. As shown in the table, the higher dropout rate of 0.9 provides better Sensitivity, Specificity, and Accuracy, indicating improved overall recognition capability.

**Table 4:** Performance Comparison with Different Dropout Rate^a^

dropout	sensitivity	specificity	accuracy	MCC	AUC
0	0.9269	0.9195	0.9195	0.2905	0.9696
0.1	0.8987	0.8110	0.8118	0.1745	0.9388
0.3	0.8706	0.9011	0.9008	0.2442	0.9537
0.5	0.8987	0.8872	0.8873	0.2357	0.9602
0.7	0.9283	0.9234	0.9235	0.2983	0.9745
**0.9**	**0.9747**	**0.9896**	**0.9895**	**0.6782**	**0.9868**

a The bolded row indicates the configuration selected for the final model.

### The Performance Comparison with or without RAG

In Table 5, the RAG_ProtTrans approach, which combines ProtTrans embeddings with a retrieval-augmented strategy, demonstrated significant advantages in the independent prediction of ATP-binding sites. This method achieved a sensitivity of 0.9747 and an AUC as high as 0.9868 on an independent data set, clearly outperforming the former method without RAG.

**Table 5:** Performance Comparison with and without RAG^a^

methods	sensitivity	specificity	accuracy	MCC	AUC
ProtTrans	0.8498	0.9433	0.9434	0.3181	0.9497
**RAG_ProtTrans**	**0.9747**	**0.9896**	**0.9895**	**0.6782**	**0.9868**

a The bolded row indicates the configuration selected for the final model.

These results underscore the RAG-based retrieval strategy further enhances sequence representation by extracting contextual information from protein databases. This combined strategy not only significantly improves prediction accuracy and robustness but also showcases the potential of incorporating domain-specific database knowledge to enrich sequence representation.

### The Performance Comparison with Previous Works

In our study, we introduce a validation data set of 69 RTK sequences into other prediction methods for comparison. In Table 6, our method performed well across various evaluation metrics, including sensitivity, accuracy, MCC, and specificity.

**Table 6:** Performance Comparison with Previous ATP-Binding Prediction Methods

methods	years	sensitivity	specificity	accuracy	MCC
TargetATPsite	2013	0.3826	0.9909	0.9851	0.3260
ATPBind	2018	0.2297	0.9961	0.9885	0.2857
NsitePred	2018	0.4494	0.9866	0.9814	0.3245
DELIA	2020	0.2140	0.9949	0.9873	0.2434
our method	2025	0.9747	0.9896	0.9895	0.6782

#### TargetATPsite^28^

This study employs a comprehensive computational framework to accurately predict ATP-binding sites in proteins. To capture evolutionary information, it extracts features using position-specific scoring matrices and residue evolution images. These features are then processed with sparse representation techniques to generate compact 128-dimensional feature vectors. A support vector machine with an RBF kernel serves as the classifier, optimized through grid search. To address class imbalance, the model incorporates random under-sampling combined with the AdaBoost ensemble method. Additionally, spatial clustering algorithms group predicted binding residues based on spatial proximity to identify potential binding pockets, delivering a reliable solution for ATP binding.

#### ATPbind^29^

The ATPbind method integrates sequence and structural information, including evolutionary data (PSSM), predicted secondary structure, predicted solvent accessibility, and both sequence and structure template predictors (S-SITEatp and TM-SITEatp) specifically designed to accurately predict these sites in proteins. To address the challenge of imbalanced data, the study employed a random undersampling strategy and trained support vector machines using a multimodel ensemble approach, ensuring a more precise identification of binding residues.

#### NsitePred^30^

NsitePred is an ensemble predictor designed to accurately identify nucleotide-binding residues in proteins by integrating diverse features and methodologies. It leverages amino acid sequence information, evolutionary profiles from PSI-BLAST-generated PSSMs, predicted structural descriptors, residue conservation metrics, and collocated amino acid pairs. Using a sliding window of size 17 to capture local context, the approach employs a support vector machine with a one-against-the-rest strategy, enhanced through wrapper-based feature selection, and combines its machine learning predictions with BLAST-based alignment results into a consensus model.

#### DELIA^31^

DELIA utilizes a combination of bidirectional LSTM networks to process sequential data and residual neural networks to handle spatial data, with grouped features enhancing overall efficiency. Additionally, the method addresses data imbalance through undersampling, oversampling, and a stacked ensemble technique that integrates multiple classifiers using logistic regression. These strategies enhance its ability to effectively handle complex biological data sets for protein–ligand binding prediction.

The comparative results presented in Table 6 highlight that our method achieves significantly superior performance in sensitivity (0.9747), accuracy (0.9895), and MCC (0.6782) relative to related previous methods. This performance advantage underscores the efficacy and robustness of our proposed approach, particularly in accurately identifying subtle binding site characteristics.

## Conclusion

This study introduces RTK_RAG, a novel computational framework that significantly enhances the prediction of ATP binding sites in receptor tyrosine kinases (RTKs). By innovatively integrating retrieval-augmented generation (RAG) with protein language models (PLMs) and multiwindow convolutional neural networks (MCNN), RTK_RAG overcomes the limitations of general predictors by specifically capturing the diverse characteristics of RTK families.

When evaluated on an independent RTK data set, RTK_RAG demonstrated superior performance, achieving an AUC of 0.9868 and a sensitivity of 0.9896. Comparative analyses with existing methods further confirm that RTK_RAG either outperforms or remains highly competitive, clearly highlighting the benefit of this specialized architecture and integration of domain-specific information. This marked improvement highlights the efficacy of our specialized architecture and the integration of domain-specific knowledge. RTK_RAG not only provides a more accurate tool for identifying ATP binding sites crucial for cancer research and therapeutic development but also offers a generalizable framework adaptable for functional site prediction in other challenging protein families. Such computational strategies are particularly valuable for developing targeted therapies against RTK-driven diseases, including cancer, and can help overcome challenges associated with drug resistance arising from mutations or altered binding site conformations.^32,33^ This work underscores the power of hybrid computational methods to advance protein function prediction, and the promising results and broader applicability also exemplify its potential to foster more precise and personalized approaches in biomedical research and clinical applications.

## Key Points

1. RTK_RAG aims to identify ATP-binding sites in receptor tyrosine kinases (RTKs), addressing the shortcomings of general ATP-binding site predictors that often overlook family features.
2. Integrate retrieval and augmented techniques to extract essential biological feature embeddings from pretrained protein language models to enrich RTK-specific information.
3. On independent RTK data sets, RTK_RAG achieves outstanding predictive performance with an AUC of 0.9868 and a sensitivity of 0.9747, significantly surpassing general ATP-binding predictors.
4. RTK_RAG’s approach establishes a generalizable framework for functional site prediction across other specialized protein families, demonstrating broad applicability beyond RTKs.
5. By supplying a more precise predictive tool, RTK_RAG facilitates drug design and potentially enhances the understanding of RTK-mediated disease mechanisms.

## Supplementary Material



## Data Availability

The authors confirms that the code and data resources supporting this study are available at: https://github.com/B1607/RTK_RAG.

## Notes

Performance evaluation metrics (Section S1): Comprehensive definitions of sensitivity, specificity, accuracy, MCC, along with their respective calculation formulas (eqs S1–S4) (PDF)
