# Supplementary material for: RTK_RAG: Leveraging Retrieval Augmented Generation with Multi-Window Convolutional Neural Networks for Superior ATP Binding Site Prediction in Receptor Tyrosine Kinases
Source: J Chem Inf Model. 2025 Jun 18;65(13):7277–84. doi: 10.1021/acs.jcim.5c00766 (PMC12264935; doi:10.1021/acs.jcim.5c00766)
Supplement: Supplementary file 1 [file ci5c00766_si_001.pdf]

SUPPORTING INFORMATION

RTK_RAG: Leveraging Retrieval Augmented Generation with Multi-Window Convolutional Neural Networks for Superior ATP Binding Site Prediction in Receptor Tyrosine Kinases

Sin-Siang Wei[1※], Wei-En Jhang[1※], Yu-Chen Liu[1], Cheng-Che Chuang[1], Yu-Yen Ou[1,2*]

[1] Department of Computer Science and Engineering, Yuan Ze University, Chung-Li, 32003, Taiwan
[2] Graduate Program in Biomedical Informatics, Yuan Ze University, Chung-Li, 32003, Taiwan

※These two authors contributed equally to this work

*Corresponding author: yien@saturn.yzu.edu.tw

Section S.1 Performance Evaluation

Once trained, the model's predictive performance was assessed using standard evaluation metrics, including sensitivity, specificity, accuracy, the Matthews correlation coefficient (MCC), and the area under the receiver operating characteristic curve (AUC). These metrics rely on four fundamental outcomes: True Positives (TP), True Negatives (TN), False Positives (FP), and False Negatives (FN), which collectively account for all possible outcomes under prediction. In other words, for each prediction, the sum of TP, TN, FP, and FN is the length of the input protein sequence.

Sensitivity, also referred to as recall, measures the proportion of true positive cases (ATP-binding residues) correctly identified by the model and was particularly crucial for evaluating the model's ability to identify ATP-binding residues accurately. While specificity quantifies the proportion of true negative cases (non-binding residues) accurately classified, accuracy evaluates the overall proportion of correct predictions across all classes. MCC provides a balanced measure of performance, with values ranging from -1 (complete disagreement) to +1 (perfect agreement), and 0 indicating random predictions. AUC, calculated as the area under the ROC curve, measures the model's ability to distinguish between positive and negative cases across varying classification thresholds. An AUC of 1 represents perfect discrimination, while 0.5 indicates random classification. The evaluation of our model's performance in predicting ATP-binding sites was rigorous, utilizing these metrics to comprehensively assess their capabilities. Among these, AUC was the primary evaluation criterion due to its robustness in summarizing the model's overall performance across multiple threshold settings.

$$Sensitivity = \left(\frac{TP}{TP+FN}\right) \quad (1)$$

$$Specificity = \left(\frac{TN}{TN+FP}\right) \quad (2)$$

$$Accuracy = \left(\frac{TP+TN}{TN+FP+TP+FN}\right) \qquad (3)$$

$$MCC = \left(\frac{(TP*TN)-(FP*FN)}{\sqrt{(TP+FP)(TP+FN)(TN+FP)(TN+FN)}}\right) \qquad (4)$$
